# Implementing Vancomycin Population Pharmacokinetic Models: An App for Individualized Antibiotic Therapy in Critically Ill Patients

**DOI:** 10.3390/antibiotics12020301

**Published:** 2023-02-02

**Authors:** Manuel Mena, Julio-Cesar Garcia, Rosa-Helena Bustos

**Affiliations:** Department of Clinical Pharmacology, Evidence-Based Therapeutics Group, Faculty of Medicine, Campus del Puente del Común, Universidad de La Sabana and Clínica Universidad de La Sabana, Km. 7, Autopista Norte de Bogotá, Chía 1400132, Cundinamarca, Colombia;manuelmega@unisabana.edu.co (M.M.); julio.garcia@unisabana.edu.co (J.-C.G.)

**Keywords:** vancomycin, Bayesian prediction, population pharmacokinetics, personalized dosing, individualized therapy, critical ill patients, therapeutic drug management, Shiny application

## Abstract

In individualized therapy, the Bayesian approach integrated with population pharmacokinetic models (PopPK) for predictions together with therapeutic drug monitoring (TDM) to maintain adequate objectives is useful to maximize the efficacy and minimize the probability of toxicity of vancomycin in critically ill patients. Although there are limitations to implementation, model-informed precision dosing (MIPD) is an approach to integrate these elements, which has the potential to optimize the TDM process and maximize the success of antibacterial therapy. The objective of this work was to present an app for individualized therapy and perform a validation of the implemented vancomycin PopPK models. A pragmatic approach was used for selecting the models of Llopis, Goti and Revilla for developing a Shiny app with R. Through ordinary differential equation (ODE)-based mixed effects models from the mlxR package, the app simulates the concentrations’ behavior, estimates whether the model was simulated without variability and predicts whether the model was simulated with variability. Moreover, we evaluated the predictive performance with retrospective trough concentration data from patients admitted to the adult critical care unit. Although there were no significant differences in the performance of the estimates, the Llopis model showed better accuracy (mean 80.88%; SD 46.5%); however, it had greater bias (mean −34.47%, SD 63.38%) compared to the Revilla et al. (mean 10.61%, SD 66.37%) and Goti et al. (mean of 13.54%, SD 64.93%) models. With respect to the RMSE (root mean square error), the Llopis (mean of 10.69 mg/L, SD 12.23 mg/L) and Revilla models (mean of 10.65 mg/L, SD 12.81 mg/L) were comparable, and the lowest RMSE was found in the Goti model (mean 9.06 mg/L, SD 9 mg/L). Regarding the predictions, this behavior did not change, and the results varied relatively little. Although our results are satisfactory, the predictive performance in recent studies with vancomycin is heterogeneous, and although these three models have proven to be useful for clinical application, further research and adaptation of PopPK models is required, as well as implementation in the clinical practice of MIPD and TDM in real time.

## 1. Introduction

The population response to therapy is the essential target of individualized medicine. In this regard, the Bayesian approach is a method that allows clinical pharmacologists to make predictions about the concentration of a drug, adjust doses and achieve therapeutic range more efficiently than in normal practice [1].

The bedside Bayesian-guided personalized dosing of vancomycin is proven and has increased the proportion of patients achieving target AUC24 (area under curve in 24 h) and the percentage time in the acceptable range (%TTR) [2]. To predict the concentration of drugs, the challenge is determining the right model that describes the compartmental profile, the changes in the clearance (Cl) and the volume of distribution (Vd) that affect the concentration of the drug in patients with impaired physiological function such as the critically ill patients, and this objective is more important for hydrophilic antibiotics such as beta-lactams, glycopeptides or aminoglycosides and drugs that can cause damage to the brain, hematological and kidney tissues [3,4,5].

In clinical practice, to prevent injuries and improve outcomes, therapeutic drug monitoring (TDM) is the recommended approach, especially for dose adjustment of narrow-therapeutic-index (NTI) drugs in critically ill patients. TDM should be implemented particularly for glycopeptides, aminoglycosides, beta-lactams and linezolid and is globally rising in popularity because it enhances results and reduces adverse events with vancomycin, especially the computerized TDM in the middle-income setting [3,5,6,7,8]. For vancomycin, TDM is established by the trough concentration, and it is recommended that the samples be taken before the fourth dose.

Nevertheless, it has been found that up to 61.5% of trough samples can be incorrectly taken, leading to additional sampling or causing delayed or wrong dose adjustment [9]. Trough concentration ranges of 10–15 mg/L in intermittent therapy for susceptible microorganisms and 15–20 mg/L for Staphylococcus aureus are used to approximate AUC. However, trough concentrations correlate poorly with AUC24 and are no longer recommended due to an association with higher AKI (acute kidney injury) incidence and lack of data to show that this higher target improves the efficacy of vancomycin. The guideline proposed by the Infectious Diseases Society of America (IDSA) and a recent position paper on critically ill patients recommends that the daily AUC24/MIC for an MIC (minimum inhibitory concentration) of 1 mg/L should be maintained for between 400 and 600 h to maximize efficacy, with AUC24 < 700 mg/L·h to minimize the likelihood of nephrotoxicity [3,10,11,12,13,14,15,16,17,18].

To optimize antimicrobial dosing and enhance the chance of reaching the pharmacokinetics and pharmacodynamics (PK/PD) target and clinical outcomes, we need to change the traditional fixed dose method (mg/day) or dose based on weight (mg/kg), supplied by standard product information, to an individualized dose strategy guided by population PK (PopPK) models for Bayesian forecasting and TDM [19,20,21,22,23,24].

Bedside individualized dosing of vancomycin increases the proportion of patients achieving target AUC24 and the %TTR, and a dosing decision support tool can be implemented and used in the clinical setting with appropriate training to maximize the efficacy and minimize the toxicity of vancomycin. Unfortunately, Bayesian-guided software is generally not available, and therefore, monitoring the AUC from the bedside is extremely difficult, and only a small percentage of practitioners implement this monitoring. Additionally, the TDM has practical limitations due to gaps in the time of administration, the time of monitoring results and the adjustment time; hence, in the majority of patients, personalized dosing starts late [2,5,25]. Moreover, the implementation of Bayesian dosing into medical practice is necessary but is limited by the complexity of mathematics, being computationally intensive; the need for specialized software; and the need for specialized knowledge for the interpretation of the PK/PD, which is affected by specific clinical conditions, immune system functionality, concomitant disorders and comedication. In fact, reducing the gaps in knowledge about antimicrobial resistance, the proper use of antibiotics and improving the availability of data for the development of tools are the main recommendations due to the growing antimicrobial resistance across the world and in Colombia [26].

Model-informed precision dosing (MIPD) is an approach to integrating different sources of information into a mathematical framework that has the potential to streamline the TDM process and maximize the success of antibacterial therapy. MIPD is a growing area of research, trying to justify its implementation in clinical practice, to enhance the flexibility and precision of dose individualization by changing either the maintenance dose or the dosing interval to keep the drug concentration in the therapeutic range. However, this approach requires model validation and re-evaluation of existing workflows and the adaptation of new, minimally invasive and noninvasive technologies based on biosensors or traditional methods to determine the concentration of drugs in biological matrices, e.g., immunoassays, high-performance liquid chromatography (HPLC) with fluorescence or ultraviolet detection and liquid chromatography with tandem mass spectrometry detection (LC-MS/MS) [5]. The MIPD and TDM implementation in clinical practice needs more information on targets, as well as education and research on PK/PD models of antibiotics, biomarkers for treatment response and clinical outcome parameters and should, through biosensor knowledge, achieve the development of rapid bioanalytical techniques for real-time TDM [27]. The portable bioanalytical systems provide laboratory testing at the site of treatment, economic feasibility and ease-of-use, with advantages over the classical immunoserological methods and modern diagnostic platforms that are expensive, require long testing time and are not always available in low-complexity hospitals and with limited economic resources [28,29]. Biosensors and the advancement of nanotechnology in the context of sepsis can offer advantages for the early detection of pathogens [30,31] and, for antibiotics such as vancomycin, the precise measurement may help overcome bacterial resistance and reduce the risk of adverse effects [32,33,34,35,36].

MIPD is based on population models, composed of (i) the structural model of the antibiotic in a patient population; (ii) a covariate submodel such as body weight, age, organ function markers or co-medication; and (iii) a mathematical representation of the interindividual and intraindividual variability of the PK parameters and residual variability around the individually predicted drug concentration. This model can be utilized before the administration of the first dose to predict a dosing regimen that maximizes the chance of meeting the PK/PD/toxicity targets. The election of an adequate model that represents the patient characteristics is one determinant for the pharmacotherapy of antimicrobials [5,37].

Shiny App is an interactive web application for users to learn more about the impact of priors and the need for sensitivity analysis in empirical situations. This app allows users to examine the impact of various prior distribution settings on final model results, such as weight or kidney function, ensuring that the user is fully aware of the substantive impact of prior selection.

The objectives of this publication were to present a platform based on open source to encourage collaborative development and to make a preliminary analysis with retrospective data from our population on the predictability of trough concentrations by PopPK models in critically ill patients.

## 2. Results

Of the cases studied, there were six women and nine men with a mean age of 53.67 years, mean weight of 68.87 kg and mean serum creatinine concentration of 0.76 mg/dL. The mean creatinine clearance obtained using the models of Llopis et al. and Revilla et al. [38,39] calculated based on Cockcroft–Gault was 106.79 mL/min, while in the study by Goti et al. [40], the mean was 95.5 mL/min.

The mean value of the volume of distribution (Vd) in the study by Revilla et al. was 70.32 L. The models of Llopis et al. and Goti et al. have significant differences in the average of the calculated Vc and Vp, since in the first model the Vp has a higher proportion (Vc: 28.51 L; Vp: 90.9 L), while in the second the Vp is fixed (Vc: 79.67; Vp: 38.4). Vancomycin clearance values calculated for the models of Llopis et al., Revilla et al. and Goti et al. were similar, with averages of 4.66 L/h, 4.32 L/h and 3.69 L/h, respectively.

In the Shapiro–Wilk test, it was found that only the distribution of the total concentration and the predictions of the Goti model showed normal behavior, while the other estimates and predictions did not present a normal distribution.

The average of the total concentration at 36 h was 15.52 mg/L (W = 0.93968, *p*-value = 0.3783), similar to the means of the estimates in the models of Revilla and Llopis, which were 15.76 mg/L (W = 0.75714, *p*-value = 0.00109) and 15.57 mg/L (W = 0.84333, *p*-value = 0.01399), respectively. In the estimates in the Goti model, a mean of 9.21 mg/L (W = 0.81623, *p*-value = 0.005984) was obtained. However, with the Wilcoxon test, there were no significant differences between the estimates and the trough concentration, (Llopis W = 154 and *p*-value = 0.08919; Revilla W = 114, *p*-value = 0.9674, Goti, W = 109, *p*-value = 0.9025).

**Figure 1:**
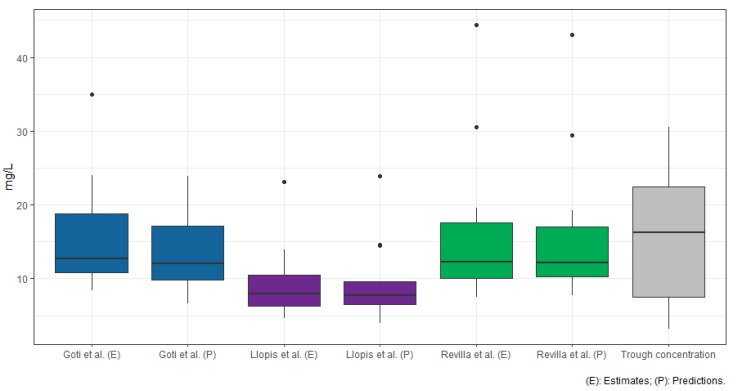
Boxplot for estimate predictions and trough concentration.

For the predictions, the means did not vary significantly from the estimates: Llopis et al. 9.26 mg/L (W = 0.8001, *p*-value = 0.003687), Revilla et al. 15.5 mg/L (W = 0.7533, *p*-value = 0.0009826) and Goti et al. 13.87 (W = 0.90782, *p*-value = 0.1254), and with Wilcoxon test, there were no significant differences either (Llopis et al. W = 154, *p*-value = 0.08919, Revilla et al., W = 117, *p*-value = 0.8702, Goti et al., W = 119, *p*-value *p* = 0.8063). In the box plot of Figure 1, it can be seen that the models of Revilla et al. and Goti et al. show similar behavior in the estimates and predictions with respect to the trough concentration, and although the Llopis model has a lower mean and grouping, it is still within the limits of the trough concentration. However, it is necessary to point out that the homogeneity between the distribution of estimates, predictions and concentration does not necessarily reflect on the performance.

Although the performance of the estimates is similar for the three models, the model of Llopis et al., which underestimates the concentration, shows a little more precision and less variation (mean 83.94%, SD 54.65%) than the Revilla et al. model (mean 136.88%, SD 81.17%), and the Goti et al. model (mean 143.6%, SD 93.92%) overestimates the minimum concentration. However, the Llopis et al. model showed a higher negative bias (mean −34.47%, SD 63.38%) and a higher RMSE (mean 10.69 mg/L, SD 12.81 mg/L), while the models of Revilla et al. (mean 10.61%, SD 66.37%) and Goti et al. (mean 13.54%, SD 64.93%) performed better on the mean bias, albeit with greater variability.

The RMSE of Revilla et al. (mean 10.65 mg/L, SD 12.81 mg/L) was similar to that of the model of Llopis et al. (mean 10.69 mg/L, SD 12.23 mg/L), while the Goti et al. model showed the lowest RMSE (mean 9.06 mg/L, SD 9 mg/L). The summary of the results is presented in Table 1.

**Table 1:** Comparative results of PopPk.

	Llopis	Revilla	Goti
	Mean (SD)		
Age (years)	**53.67** (23.53)		
Weight (Kg)	**68.87** (14.63)		
Creatinine (mg/dL)	**0.76** (0.19)		
Clcr (mL/min) *	**106.79** (41.63)		**95.5** (42.27)
Vc (L)	**28.51** (6.06)	**70.32** (43.96)	**79.67** (35.26)
Vp (L)	**90.9** (19.32)		**38.4** (0)
Cl (L/h)	**4.66** (1.52)	**4.32** (1.67)	**3.69** (1.34)
**Trough concentration (mg/L)**	**15.52** (9.24)		
**Estimated**	**9.21** (4.74)	**15.76** (9.92)	**15.57** (7.15)
Accuracy (%)	**83.94** (54.65)	**136.88** (81.17)	**143.6** (93.92)
Bias (%)	**−34.47** (63.38)	**10.61** (66.37)	**13.54** (64.93)
RMSE (mg/L)	**10.69** (12.81)	**10.65** (12.23)	**9.06** (9)
**Predicted (mg/L)**	**9.26** (5.09)	**15.5** (9.49)	**13.87** (5.55)
Accuracy (%)	**80.88** (46.5)	**136.02** (81.57)	**123.68** (75.22)
Bias (%)	**−35.11** (59.73)	**9.78** (66.64)	**3.79** (57.42)
RMSE (mg/L)	**10.63** (6.65)	**10.46** (11.83)	**7.46** (8.81)

* The difference in Clcr is due to the fact that the Goti model truncates creatinine when calculating for the elderly and if it is greater than 150 mL/min, see Table 2.

**Table 2:** Model of population pharmacokinetic parameters of vancomycin chosen for our study.

	Llopis et al. [38]	Revilla et al. [39]	Goti et al. [40]
Fixed parameters	$Cl=\theta_{1}\times Cl_{cr}+\theta_{2}\times TBW$ $\theta_{1}=0.034$ $\theta_{2}=0.015$ $V_{c}=\theta_{3}\times TBW$ $\theta_{3}=0.414$ $Q=\theta_{4}$ $\theta_{4}=7.48$ $V_{p}=\theta_{5}\times TBW$ $\theta_{5}=1.32$	$Cl=\theta_{1}\times Cl_{cr}+AGE^{\theta_{2}}$ $\theta_{1}=0.67$ $\theta_{2}=-0.24$ $V=\theta_{3}\times \theta_{4}^{A}$ $\theta_{3}=0.82$ $\theta_{4}=2.49$ A= 0 if Cr ≤1 mg/dL A= 1 if Cr>1 mg/dL	$Cl=\theta_{1}\times {\left(\frac{Cl_{cr}}{120}\right)}^{\theta_{2}}\times \theta_{3}{}^{DIAL}$ $\theta_{1}=4.5$ $\theta_{2}=0.8$ $\theta_{3}=0.7$ $V_{c}=\theta_{4}\times \left(\frac{TBW}{70}\right)\times \theta_{5}{}^{DIAL}$ $\theta_{4}=58.4$ $\theta_{5}=0.5$ Q=6.5 $V_{p}=38.4$ DIAL= 1 on dialysis DIAL= 0 without dialysis Clcr= 150 mL/min if Clcr>150 mL/min Scr=1 mg/dL if Scr<1 mg/mL and age>65 years
Interindividual variability (CV%)	$\omega_{Cl}=29.2$ $\omega_{V_{c}}=36.4$ $\omega_{V_{p}}=39.8$	$\omega_{Cl}=30.13$ $\omega_{V}=22.83$	$\omega_{Cl}=39.8$ $\omega_{V_{c}}=81.6$ $\omega_{V_{p}}=57.1$
Intraindividual variability (SD mg/L)	$\sigma_{1}=4.88$ $\sigma_{2}=4.3$	$\sigma_{1}=4.23$	$\sigma_{1}=5.13$ $\sigma_{2}=3.4$

*Cl*: vancomycin clearance (L/h); *Cl_cr_*: creatinine clearance (mL/min); TBW: total body weight (kg); *Scr*: serum creatinine (mg/dL); *V_c_*: volume of the central compartment (L); *V_p_*: volume of the peripheral compartment (L); *V*: distribution volume (L); *ω_Cl_*: interindividual variability of clearance; *ω_Vc_*: interindividual variability of the central compartment; *ω_Vp_*: interindividual variability of the peripheral compartment; *ω_V_*: interindividual variability of the distribution volume.

**Figure 2:**
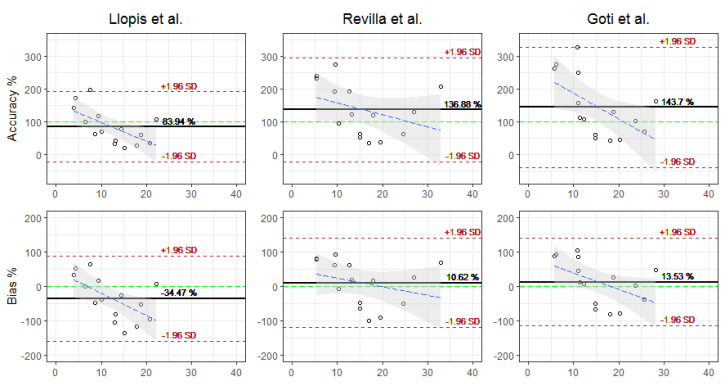
Bland–Altman plot for estimates.

**Figure 3:**
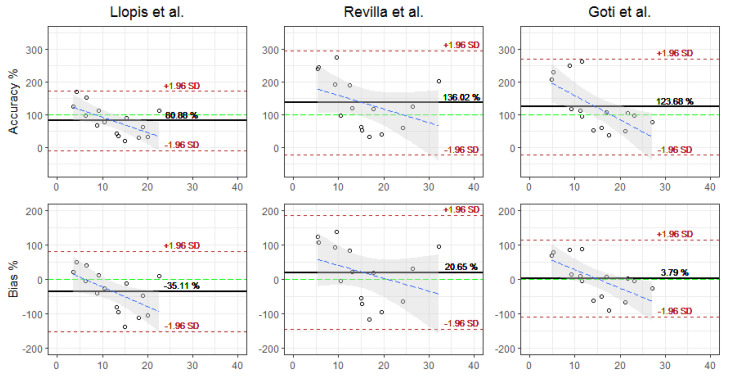
Bland–Altman plot for predictions.

For the predictions, the performance results did not differ significantly and showed the same behavior as the estimates. The precision of the model of Llopis et al. had a mean of 80.88% and an SD of 46.5%. Revilla et al. had a mean of 136.02% and an SD of 81.57%, and Goti et al. had a mean of 123.68% and an SD of 75.22%. The bias was similar, Llopis et al. had a mean of −35.11% and an SD of 59.73%, Revilla et al. had a mean of 9.78% and an SD of 66.64%, and Goti et al. had a mean of 3.79% and an SD of 57.42%. The RMSE did not differ significantly either, Llopis et al. had a mean of 10.63 mg/L and an SD of 6.65 mg/L, Revilla et al. had a mean of 10.46 mg/L and an SD of 11.83 mg/L, and Goti et al. had a mean of 7.46 mg/L and an SD of 8.81 mg/L. Figure 2 and Figure 3 show the Blant–Almant graphs, where it can be seen that the model of Llopis et al. is the one with the best performance with the lowest correlation margins. The Goti et al. model is the model that follows in performance, although with similar correlation margins to the Revilla et al. All the plots showed a tendency to underestimate or underpredict with respect to concentrations greater than 10 mg/L for the Llopis model and 15 mg/L for the Goti and Revilla models. Meanwhile, for lower concentrations, the data may be more grouped, since as shown by Figure 1, the mean of the calculations of the estimates and predictions for all the models was below the mean of the concentrations measured in the patients.

## 3. Discussion

Although we did not intend to carry out a rigorous external validation, this is the preliminary validation to determine whether there is an adequate implementation of the models and to be able to move toward an adaptation process through the continuous learning approach. It is assumed that, in clinical practice, the challenge is to create a target-concentration-controlled infusion (TCI) strategy, as an infusion method for personalized patient dosing in critically ill patients; however, the PK model must undergo adequate external validation [41]. Commonly available Bayesian pharmacokinetic models lack precision in the critically ill; until this precision is improved, AUC24 calculation using two concentrations via kinetic equations or continuous vancomycin infusion with a single concentration at steady state may be preferable in this population [42]. In a recent study for vancomycin, the predictive performance was very heterogeneous across the 31 evaluated models, and the calculated PK/PD target attainment in AUC24/MIC calculation differed by more than threefold across models, which would impact dosing decisions [19]. The poor performance when the models are translated to a new patient population requires the use of methods such as (i) the continuous learning approach to adapt a population model to a local environment, which reduced the prediction error by 2–13% compared with previous models for pediatric patients treated with vancomycin [43], and (ii) the automated model averaging/selection approach recently studied for vancomycin models, which uses a number of candidate models, some of which may be specified for a patient [44].

In the first review of population pharmacokinetic analyses, only two models could accurately describe vancomycin pharmacokinetics in all populations [45]. In a study conducted in 2013 in Chinese adult patients, Purwonugroho et al. [46]. and Llopis-Salvia et al. [38] had better predictive performance [47]. In a review that included 30 PopPK analyses on vancomycin, most of the studies aimed at developing a PopPK model in a special subpopulation to determine the PK profile and PK parameters that are key for the optimization of vancomycin dosage regimens [48]. In an external evaluation of population pharmacokinetic models of vancomycin in large cohorts of intensive care unit patients from Amsterdam, the models of Llopis-Salvia et al. and Revilla et al. [39] showed acceptable bias but low accuracy and showed relatively low root mean square error (RMSE) while showing high inaccuracy [49]. More recently, an evaluation of the predictive performance of pharmacometrics models in critically ill patients conducted in Australia showed that the models of Goti et al. [40], Llopis et al. [38] and Roberts et al. [50] are clinically appropriate to inform vancomycin dosing in critically ill patients, and the Goti model was the only clinically acceptable model with both a priori (rBias 3.4%) and Bayesian forecasting (rBias 1.5%) approaches [20]. The external evaluation should be the first step in a pharmacokinetic analysis of vancomycin; for this purpose, from the Sabana University Clinical Pharmacology Department, within the antibiotic stewardship strategy, we developed an application that uses population pharmacokinetic models in order to improve skills in precision medicine, explore the MIPD-based approach for antibiotic management and prove that the use of linear estimators and Bayesian approaches are comparable, since recent studies have shown that the two-concentration linear and Bayesian methods demonstrated high-level agreement with acceptable variability and can be considered comparable for estimating the AUC24 of vancomycin [51].

Although there are no significant differences between the results of the three models, there are differences between the approaches, for example, the Goti model may have better performance because it takes into account the truncation of the creatinine clearance (Clcr) or the serum creatinine (Scr), since there is a significant correlation between increased renal clearance (ARC) and lower vancomycin trough serum concentrations (MCV) during therapy [52]. Furthermore, the MIPD platform must be tested on the platform’s usability, performance and adaptability to the clinical environment and special conditions of patients. Precisely, the advantage of MIPD is that it captures drug, disease and patient characteristics in modeling approaches and can be used to perform Bayesian forecasting and dose optimization [27]. Recently, a consensus review by the Japanese Society of Chemotherapy and the Japanese Society of Therapeutic Drug Monitoring promoted MIPD for vancomycin, developed statements for TDM and provided recommendations based on MIPD to increase treatment response while preventing adverse effects and also recommended expanding the use of AUC-guided dosing and the availability of verified open and free Bayesian dose-optimizing software programs [53].

Bayesian-guided monitoring is always faster and more reliable than equation-guided monitoring in pre-steady-state dosing intervals in the absence of a loading dose [54]. There are five Bayesian dose-optimizing software programs that can be used for vancomycin (Adult and Pediatric Kinetics (APK), Best-Dose, DoseMe, InsightRx and Precise PK) that have been compared for the prediction of AUC with two measurements of concentrations and produce similar estimates; although their use has advantages, it requires the purchase of software and additional training [55]. Different open-source and web-based MIPD platforms for vancomycin have been presented globally, such as VancoCalc from Canada [56], TDMx from Germany [57], PATver1.1 from Japan [58] and a draft version of Vancomycin TDM from Korea [59], which have been able to demonstrate their potential benefit for application in clinical practice, although limited at the moment for academic approaches. Furthermore, in regards to TDM, there are key barriers such as the uncertainty of service processes and accessibility of dose advice and, a novel domain, ‘Trust’ [60].

No precise data were found on the sampling times and infusion periods, which were assumed to be in accordance with the established clinical protocol. This could be considered as a limitation of the study.

## 4. Materials and Methods

### 4.1. Selected Structural Models

The models were selected by a pragmatic approach, for academic proposals with minimum of models that provide in the interface the ability to compare compartmental modeling approaches that were relevant to the literature review. Llopis et al. and Goti et al. [38,40] are two-compartment models, which differ in that the Goti model truncates the creatinine when calculating Clcr in the elderly and truncates the value if it is greater than 150 mL/min, both of which have been shown to be clinically appropriate to inform vancomycin dosing in critically ill patients [19], and the Goti model is also suitable for improving precision dosing in hospitalized patients [20]. The Revilla et al. model [39], which is one-compartment model, is easier to teach, although its clinical implementation has not been satisfactorily tested. Table 2 summarizes the models.

### 4.2. App Development

**Figure 4:**
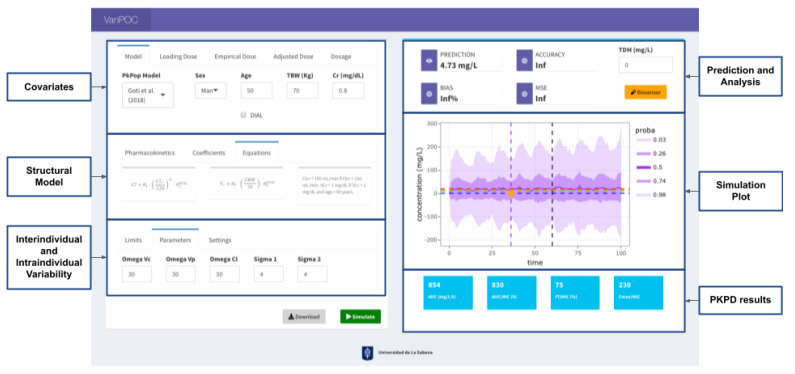
Design of Shiny App.

A Shiny app was developed with R software (v. 4.2) (Indiana, USA) (Figure 4). This app was divided into two modules: On the left side is the module to register the covariates, establish the structural model, the dosage regimen and the simulation parameters such as the interindividual and the intraindividual variability. On the right side is the module where the simulation is carried out by the mlxR package with the Monolix-Lixoft application programming interface (API). The PopPK models computed using the pkmodel() function are presented in the Table 2, and the simulations were performed with the function simulx(), which implemented complex ODE-based mixed effects models, using the model coding language Mlxtran. The simulation plot is presented together with the results at the time of TDM, the prediction analysis and the results in the PK/PD. This application is the minimum viable product (MVP) for the implementation of an MIPD platform, as a resource in optimization strategies for the use of antibiotics, so it also has a module for connecting a potentiostat for the integration of electrochemical biosensors that develop the clinical pharmacology group for TDM in real time. Figure 4 shows the dashboard, which aims to display the information on a single screen and create a gamification-oriented interface. The open-source code is available at https://github.com/LSPOC/VanPOC (accessed on 1 January 2023).

### 4.3. Data Extraction

The retrospective data came from the electronic medical records (EHRs) of adult patients admitted to the intensive care unit (ICU) of the La Sabana Clinic between December 2021 and June 2022. These patients received vancomycin with therapeutic follow up and did not receive renal replacement therapy. No particular clinical condition was considered to be selected, and they were chosen arbitrarily from the list of patients registered by the Antimicrobial Use Optimization Programs. The necessary recorded covariates for each case for all models were sex, age, weight, creatinine concentration (mg/dL) and also, for the simulation, the ordered formulation scheme, loading dose, maintenance dose, interdose interval and infusion time. Finally, for the predictive performance evaluation, the trough concentration was collected before the fourth dose (36 h) measured by an immunoassay.

### 4.4. Models’ Evaluation

The models were evaluated with a total of 15 patients. The inputs are the covariates and ordered formulation scheme for each case, which were loaded and simulated in the app. We define as estimate trough concentration at 36 h, the calculated trough concentration with the fixed-parameter models presented in Table 2, without interindividual and intraindividual variability. For predictions, which add a random effect at the same time, a CV of 30% for interindividual variability and an SD of 3 mg/L for intraindividual variability were arbitrarily defined, but they were close to those presented on the proportional and additive models used by the studies. By default, with the simulx() function, the app simulates 1000 replicates and returns a result with a 95% confidence interval. From five predictions obtained, the median was chosen to perform the analysis, because each time a simulation is run with these variability conditions it will give a different result. For each case (***j***), the predictive performance of the concentration estimated and predicted by the simulation (***C_simj_***) was evaluated by contrast with the collected retrospective data of trough concentration before the fourth dose (***C_obsj_***), using mean accuracy, mean bias and ***RMSE***, which were calculated according to Equations (1)–(3). The mean accuracy and mean bias were formulated following the review by Giavarina [61] for Bland–Altman analysis. The accuracy is represented as a ratio converted to percentage and the bias as the percentage difference with respect to the mean between the ***C_simj_*** and the ***C_obsj_***.

(1) $$\bar{Accuracy}=\frac{1}{n}\overset{n}{\underset{j=1}{\sum }}\frac{\;C_{simj}}{C_{obsj}}\times 100\%$$

(2) $$\bar{Bias}=\frac{1}{n}\overset{n}{\underset{j=1}{\sum }}\frac{\;C_{simj}-C_{obsj}}{\left(C_{simj}+C_{obsj}\right)/2}\times 100\%$$

(3) $$RMSE=\sqrt{\frac{1}{n}\overset{n}{\underset{j=1}{\sum }}{\left(C_{simj}-C_{obsj}\right)}^{2}}$$

### 4.5. Statistical Analysis

All analyses were performed using the R software (Indiana, USA) language. The mean, standard deviation and interquartile ranges of the characteristics of the evaluated population were calculated. Estimates, predictions, trough concentration and model run results were obtained. The normal distribution of the parameters was evaluated using the Shapiro–Wilk test. An analysis of mean differences was performed using the Wilcoxon test, and the visual interpretation was presented through a box plot. Finally, the performance results were presented in Bland–Altman plots.

## 5. Conclusions

The preliminary results are acceptable for the implementation of the models in the app and for translation to our usual clinical practice, but it is necessary to delve into the performance aspects that have not yet been tested with prospective data and an experimental design that allows systematic errors to be eliminated. The platform that was developed is intended to be applied in MIDP, in addition to engaging in the TDM process, with the adaptation of biosensors to clinical work routines, which will allow a precise approach to infections, allowing early diagnosis or TDM in real time that can be used to adjust the predictive models.

## Data Availability

The data presented in this study are available on request from the corresponding author. The data are not publicly available because they are stored in the research system of the Therapeutic Evidence Group.
